# Stability and Influence of Storage Conditions on Nanofibrous Film Containing Tooth Whitening Agent

**DOI:** 10.3390/pharmaceutics13040449

**Published:** 2021-03-26

**Authors:** Siriporn Okonogi, Adchareeya Kaewpinta, Pisaisit Chaijareenont

**Affiliations:** 1 Department of Pharmaceutical Sciences, Faculty of Pharmacy, Chiang Mai University, Chiang Mai 50200, Thailand; 2 Research Center of Pharmaceutical Nanotechnology, Chiang Mai University, Chiang Mai 50200, Thailand; pisaisit.c@cmu.ac.th; 3 Interdisciplinary Program in Nanoscience and Nanotechnology, Faculty of Science, Chiang Mai University, Chiang Mai 50200, Thailand; adchareeya_k@cmu.ac.th; 4 Department of Prosthodontics, Faculty of Dentistry, Chiang Mai University, Chiang Mai 50200, Thailand

**Keywords:** carbamide peroxide, nanofibrous film, stability, stability kinetics, physicochemical properties, storage

## Abstract

Carbamide peroxide (CP), a tooth whitening agent, is chemically unstable. The present study explores stability enhancement of CP by loading in a nanofibrous film (CP-F) composed of polyvinyl alcohol/polyvinylpyrrolidone/silica mixture, using an electrospinning technique. Kept at a temperature range of 60–80 °C for 6 h, CP in CP-F showed significantly higher stability than that in a polymer solution and in water, respectively. Degradation of CP in CP-F could be described by the first order kinetics with the predicted half-life by the Arrhenius equation of approximately 6.52 years. Physicochemical properties of CP-F after long-term storage for 12 months at different temperatures and relative humidity (RH) were investigated using scanning electron microscopy, X-ray diffractometry, differential scanning calorimetry, and Fourier transform infrared spectroscopy. It was found that high temperature and high humidity (45 °C/75% RH) could enhance water absorption and destruction of the nanofibrous structure of CP-F. Interestingly, kept at 25 °C/30% RH, the nanofibrous structure of CP-F was not damaged, and exhibited no water absorption. Moreover, the remaining CP, the mechanical properties, and the adhesive properties of CP-F were not significantly changed in this storage condition. It is concluded that the developed CP-F and a suitable storage condition can significantly improve CP stability.

## 1. Introduction

Carbamide peroxide (CP) is an active ingredient for tooth whitening [1]. This compound is known as urea peroxide or hydrogen peroxide–urea. CP was first used as an anti-inflammatory and antiseptic for the treatment of periodontal diseases and gingivitis [2,3]. However, tooth whitening occurred as a side effect during the treatments [4]. The whitening action of CP resulted from a chemical oxidative process involving the peroxide and organic pigmented molecules in enamel and dentine. The change in the structure of the pigmented molecules resulted in clearer, smaller molecules, and the teeth appeared white [5,6,7]. Due to the increased interest in the esthetics of healthy white teeth, dental whitening procedures became more popular [8] and tooth whitening products containing CP are widely used. Other applications of CP in the oral cavity are for the treatment of plaque, gingivitis, and caries, by its antibacterial and anti-inflammatory activities [9,10,11].

Despite its attractive properties, CP is chemically unstable [12]. It is highly sensitive to light and thermal exposure [13]. These factors are the main cause of CP degradation upon storage and result in a reduction in tooth whitening efficacy [14]. Many stabilizers and deterioration inhibitors have been used for preventing CP degradation. However, the stabilizers are varied in their effectiveness and exhibit disadvantages such as being expensive, failing to prevent effervescence, imparting undesirable color, or lacking sufficient solubility [15]. The aqueous formulations containing tooth whitening agents show the severe disadvantage of poor stability during long-term storage [16], leading to the products losing their tooth whitening potency [17].

The development in pharmaceutical technology has made it possible to produce functional formulations to overcome drug problems such as low stability [18]. Encapsulation of a drug in a dry form of nanofibrous film with diameters in the nano range by electrospinning technique is currently gaining a large interest, due to its simplicity, capacity to produce non-woven nanofibrous film with a high surface to volume ratio, low cost, and capability of scale-up production [19,20]. The electrospun nanofibrous film is a viable formulation that can allow active compounds to be incorporated with an appropriate polymer or polymer mixture. Considering that CP is highly unstable, particularly in aqueous systems, drug delivery in terms of solid formulation such as nanofibrous film should be a good candidate for delivery of this agent. In addition, the nanofibrous film formulation can increase patient compliance due to its convenience of use [21].

Recently, we reported that CP-loaded nanofibrous film (CP-F) could be produced by electrospinning technique for tooth whitening [22]. Polyvinyl alcohol (PVA) was used as a base solution for electrospinning nanofibrous film production. Polyvinylpyrrolidone (PVP) and silica helped to stabilize the CP and were used as drug carriers for the prevention of CP degradation during the process. The developed CP nanofibrous film exhibited high drug entrapment efficacy and tooth whitening activity. However, the stability of CP in the developed CP-F has not yet been comprehensively investigated. Therefore, stability tests of this novel formulation to predict monitoring and to determine the validity and the ideal storage conditions are needed. Stability testing of formulations could provide evidence of the quality of the formulation and the influence of environmental factors, such as temperature, light, and humidity [23]. The evidence can be applied to developing a suitable manufacturing process, selecting packaging, and storage conditions. Therefore, the aim of the present study was to investigate the stability of CP in CP-F after keeping it in various conditions. The degradation kinetics were studied to estimate the half-life and shelf-life of the developed products. The physicochemical properties of CP-F were characterized and the amount of CP remaining in CP-F was determined to evaluate the efficiency of CP-F on stabilization of CP.

## 2. Materials and Methods

### 2.1. Materials

CP, PVA (molecular weight = 85,000–124,000, degree of hydrolysis = 87–89%), PVP, N, N-dimethylformamide, urea, and triphenylphosphine were obtained from Sigma Aldrich (St. Louis, MO, USA). Hydrophilic fumed silica was from Evonik (Aerosil 380F, Essen, Germany).

### 2.2. Preparation of CP-F

Preparation of CP-F was according to the procedure reported in previous work [22]. Briefly, CP solution composed of PVA, PVP, silica, CP, and water in a weight ratio of 5.5:3:1:0.5:90 was firstly prepared by dissolving PVA and PVP in distilled water, and continuously stirred at 70 °C for 12 h. The prepared PVA–PVP solution was cooled to room temperature. Silica and CP were weighed and dispersed in 1% N, N-dimethylformamide. Afterwards, the prepared PVA–PVP solution was added to this solution until the final concentration of CP was 0.5%. The sample was gently stirred until a clear solution was obtained. This CP solution was used for electrospinning. For fabrication of CP-F, the electrospinning process was performed. The setup consisted of a high-voltage power supply (FC Series Glassman High Voltage Regulated DC Power Supplies, High Bridge, NJ, USA), a syringe connected with a pump (Harvard Apparatus Pump 11 Elite Syringe Pumps, Holliston, MA, USA), and a stationary metal collector (VWR International, Radnor, PA, USA) covered with aluminum foil. The prepared CP solution for electrospinning was transferred to a syringe fitted with a stainless-steel needle (Hamilton 2.5 mL, Model 1005 TLL SYR, Hamilton Metal Hub Needles, Bonaduz, Switzerland) and was horizontally pumped at a flow rate of 10 µL/min. The electrospinning was set at 15 kV and the distance between the syringe tip and the collector plate was 10 cm. Prior to the further test, the obtained CP-F was cut into 10 mm × 50 mm and measured for thickness at 10 points using a micrometer (INSIZE 3203-25A, Suzhou, China). The thickness value was confirmed by optical microscope (Axio Vert.A1 FL-LED, ZEISS, Oberkochen, Germany) equipped with a digital camera (ZEISS Axiocam 105 color). The sample was cut in a cross-sectional direction and vertically fixed on a glass slide. Photomicrographs of the samples were examined at magnification 5× and measured for thickness by Image J software (US National Institutes of Health, Bethesda, MD, USA). CP in polymer solution (CP-P) was prepared by dissolving CP in a polymer solution containing 5.5% PVA, 3% PVP, and 1% silica to have a final CP concentration of 5%. CP in water solution (CP-W) was obtained from dissolving CP in distilled water to obtain a final concentration of CP the same as CP-P. The amount of CP was analyzed using high-performance liquid chromatography (HPLC).

### 2.3. HPLC Analysis

For the determination of CP remaining in the samples, HPLC (Hewlett Packard series 1100, Agilent Technologie, Santa Clara, CA, USA) was performed, and HPLC condition from previous reports [24] was used with some modifications. Briefly, an amount of 0.1 g of sample was dissolved in 10 mL deionized water, then the solutions were centrifuged using a Sorvall^TM^ ST16R Centrifuge (Thermo Fisher Scientific, Waltham, MA, USA) with a speed of 10,000 rpm for 15 min. An amount of 1000 μL of the collected samples was mixed with 1000 μL of 0.1 M triphenylphosphine and stirred for 2 h with light protection. The determination was carried out at 25 ± 0.2 °C. A reversed-phase column (4.6 mm × 250 mm Hypersil ODS Agilent technologies, Santa Clara, CA, USA) was used and detected at 225 nm. The injection volume was 10 μL. A mobile phase at different ratios of acetonitrile to water was run with a flow rate of 1.0 mL/min. At the starting of running time, a volume ratio of 50:50 was used until 6.5 min. After that, the mobile phase ratio was changed to 100:0. At 10 min, the mobile phase ratio was changed back to 50:50 until the complete run time of 25 min was reached. The calibration curve was prepared using an aqueous solution of CP at a range of 50–200 μg/mL. A linear standard curve was obtained with a correlation coefficient (*r*^2^) of 0.9997. The amount of CP remaining was calculated using Equation (1):

(1) $$\%\mathrm{CP}\;\mathrm{remaining}\;=\;\frac{{\mathrm{CP}}_{a}}{{\mathrm{CP}}_{i}}\;\times \;100,$$

where CP_a_ was the analyzed amount of CP at the time interval and CP_i_ was the amount of CP initially in the sample. The results obtained from all storage conditions were plotted as %drug remaining versus time.

### 2.4. Effects of Temperature and UV Light on Degradation Kinetics of CP

To investigate the thermal degradation kinetics of CP, the samples were placed in the accelerated temperature conditions of 60 °C, 70 °C, and 80 °C. Exact amounts of 20 mg of CP-F, CP-P, and CP-W were weighed in 1.5 mL microcentrifuge tubes and placed in a heating incubator (MD-MINI, major science, Saratoga, CA, USA), and protected from light. To investigate the effects of UV light, the samples were placed in a closed chamber and exposed to UV light at 254 nm using a 35 W, CKL T5 fluorescent lamp (Zhongshan Okes Lighting Appliance Co.,Ltd, Guangdong, China) at a constant temperature of 25 °C. A control was performed by placing the samples in a closed chamber without UV light exposure at the same temperature. The samples were collected after exposure times of 60, 120, 180, 240, 300, and 360 min. The remaining amount of CP was analyzed using HPLC. The experiments were performed in triplicate.

### 2.5. Effects of Temperature and Humidity on CP-F after Long-Term Storage

A long-term stability study of CP-F was carried out for a period of 12 months at three different conditions: 25 °C/30% relative humidity (RH), 25 °C/75% RH, and 45 °C/30% RH. For conditions of 30% and 75% RH, the samples were stored in desiccators equilibrated with the saturated solutions of magnesium chloride and sodium chloride, respectively. The samples were analyzed in terms of physical properties after 12 months of storage, in comparison with those of an initial preparation as a control. The remaining amount of CP at time intervals of 1, 2, 3, 6, 9, and 12 months was determined using HPLC. The experiment was carried out in triplicate for each sample in all storage conditions.

### 2.6. Color Measurement

The color of CP-F was analyzed using a colorimeter (Fru WR10 portable precision colorimeter, Shenzhen wave optoelectronics technology Co.,Ltd, Shenzhen, China). The measurements were taken from three different points on the surface of CP-F. Color measurement outcomes were evaluated under the CIE (Commission International d’Eclaraige) L*a*b* coordinate values, where L* represents the degree of lightness ranging from 0 (zero) to 100 (white), and a* and b* represent the degree of green−red and the degree of blue−yellow color coordinates, respectively [25]. A positive a* value indicates the degree of red and a negative a* value indicates the degree of green. A positive b* value indicates the degree of yellow and a negative b* value indicates the degree of blue. The center of the a* and b* coordinates is achromatic and the increasing values of a* and b* represent the saturation of the color. The L*a*b* values of CP-F were measured. To evaluate the color change between the color of CP-F initially and 12 months after storage, the total color difference (ΔE) was calculated using Equation (2). The ΔE value relates to the visual perception of color. If the ΔE values are below 1, the color change cannot be visible, if the ΔE values are 1 to 3, a minor color change is visible, and if the ΔE values are above 3, the color change is obviously visible. The color measurement was performed at 5 points from three independent samples at each storage condition.

(2) $$\Delta E\;=\;{\left[{\left(\Delta L*\right)}^{2}\;+\;{\left(\Delta a*\right)}^{2}\;+\;{\left(\Delta b*\right)}^{2}\right]}^{1/2}.$$

### 2.7. Morphology Study

The morphology of CP-F before and after storage was observed using a scanning electron microscope (SEM, JSM 5910 LV, Tokyo, Japan). The samples were cut to small sections of approximately 0.5 cm × 0.5 cm and were attached to the stubs by using double-sided carbon tape. The samples were coated with gold by a sputter coater before SEM observation. SEM images were taken at 3000 and 10,000 magnifications with an excitation voltage of 15 kV. The average diameter of CP-F was measured using Image J software (US National Institutes of Health, Bethesda, MD, USA).

### 2.8. Internal Structure Investigation

X-ray diffraction (XRD) was undertaken using a Rigaku SmartLab X-ray diffractometer (Rigaku, Tokyo, Japan). A Bragg angle (2θ) was used at a range of 10° to 60°. The samples were placed on an etched glass slide and scanned at a rate of 12°/min. For comparison, the XRD analysis of intact CP and the blank nanofibrous film was also performed.

### 2.9. Thermal Behavior Investigation

The thermal behavior of CP-F was investigated using differential scanning calorimetry (DSC, DSC 8000, PerkinElmer, Waltham, MA, USA). The samples of approximately 1–3 mg were accurately weighed, and were transferred into aluminum pans and hermetically sealed. Subsequently, the samples were heated from 0 to 250 °C at the heating rate of 10 °C/min under nitrogen flow at 40 mL/min. For comparison, the thermal analysis of intact CP and the blank nanofibrous film was also performed.

### 2.10. Molecular Interaction Study

Fourier transform infrared spectroscopy (FTIR) was performed to investigate the molecular interaction of the samples using a Thermo Nicolet NEXUS 470 FT-IR (Thermo electron corporation, Thermo Fisher Scientific, Waltham, MA, USA) connected with a Smart diffuse reflectance FTIR accessory (Thermo electron corporation, Thermo Fisher Scientific, Waltham, MA, USA). Spectra were recorded at 25 °C in the scanning range of 4000–600 cm^−1^. The transmittance mode was used with a resolution of 4 cm^−1^ and 64 scans. Data recording was done through OMNIC software (Thermo Fisher Scientific Waltham, MA, USA). For comparison, FTIR analysis of CP powder and the blank nanofibrous film was also performed.

### 2.11. Mechanical Property Investigation

Mechanical properties of CP-F were evaluated using a texture analyzer (TA.XT Plus, Texture Analyzer Stable Micro Systems, Surrey, UK) by the method previously described [26], with some modification. Prior to testing, a texture analyzer was calibrated with a 5 kg load cell and equipped with tensile grips (A/TG). CP-F was cut into a rectangular shape of 0.5 cm × 5.0 cm. The sample was clamped between the grips. The initial length between grips was set at 3 cm. The test speed was 1 mm/s with 5 g of trigger force. The sample was pulled until the breaking of the sample occurred. At the point of breaking, the value of force and elongation was recorded. The measurement was done with three independent film samples from each storage condition. The mechanical properties of the films were characterized by the tensile strength (σ), elongation at break (ε), and Young’s modulus (E), calculated by using Equations (3)–(5), respectively:

(3) $$\sigma \;=\;\frac{F}{A},$$

(4) $$\varepsilon \;=\;\frac{\Delta L}{L_{0}},$$

(5) $$E\;=\;\frac{\sigma }{\varepsilon },$$

where F is the maximum force at the films breaking (N), A is the cross-sectional area of the sample (cm^2^), ΔL is the extension of the sample, and L_0_ is the original length of the sample (cm).

### 2.12. Mucoadhesive Property Investigation

A texture analyzer (TA.XT Plus Texture Analyzer, Stable Micro Systems, Surrey, UK) was utilized to investigate the adhesive properties of CP-F using a method previously described [22], with some modification. Prior to testing, a texture analyzer was calibrated with a 5 kg load cell. CP-F was attached to the probe (P 0.5 Perspex, 0.5-inch diameter) using double-sided adhesive tape. A piece of 2 cm × 5 cm porcine intestinal mucosa was attached to a glass slide and then placed on the stand. The surface of the mucosa was hydrated by dropping 1 mL of artificial saliva. The probe was lowered to contact the mucosal surface. A contact force of 0.2 N was applied with a contact time of 60 s, and then the probe was withdrawn at the rate of 1 mm/s. The Texture Exponent software (Stable Micro Systems, Surrey, UK) was used to determine the adhesive force. The experiment was conducted in triplicate for the film samples from each storage condition.

### 2.13. Statistical Analysis

Descriptive statistics for continuous variables were calculated and expressed as mean ± standard deviation (SD). Significance was assessed by one-way analysis of variance (ANOVA) and followed by Duncan’s multiple range test, using Statistic SPSS version 22 (SPSS Inc., Chicago, IL, USA). The significance level was set at *p* < 0.05.

## 3. Results and Discussion

The purpose of stability testing is to obtain information on how the quality of a formulation varies with time under the influence of various environmental factors, such as temperature, humidity, and light. The obtained results can lead to establishing the recommended suitable storage conditions of the formulations. The stability of a drug refers to the chemical and physical integrity of the dosage and the ability of the formulation to maintain drug content. In the present study, short-term (6 h) and long-term (12 months) stabilities of CP-F were investigated, mainly to evaluate the degradation kinetics and the gradually chemical changes of CP in CP-F, respectively, as well as the physicochemical changes after long storage in constraining conditions.

**Figure 1:**
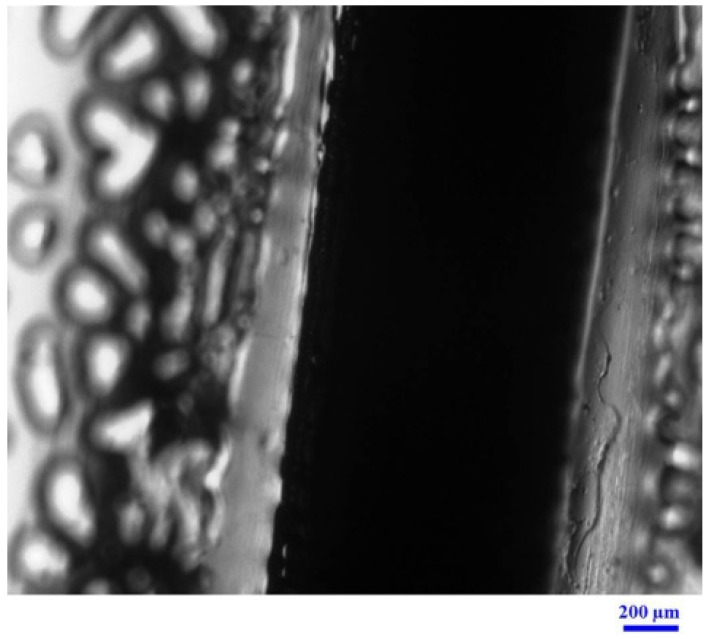
Photomicrographs from an optical microscopy of the cross-section of CP-loaded nanofibrous film (CP-F).

It was found that most fabricated CP-F had a uniform thickness. Using a micrometer, the films showed an average thickness of 0.98 ± 0.10 mm. The cross-section photomicrograph from optical microscopy of CP-F as presented in Figure 1 showed that the thickness of the films was 1.00 ± 0.05 mm, which was in accordance with the result from the micrometer. The obtained CP-F having a thickness of approximately 1 mm were selected to further study.

In general, the HPLC analysis used for the determination of CP in the formulation was validated by the selectivity of triphenylphosphine oxide and triphenylphosphine. The HPLC chromatogram peaks of triphenylphosphine oxide and triphenylphosphine were presented in the different retention times of 5.0 min and 10.5 min, respectively, as shown in Figure S1a [27]. Triphenylphosphine oxide was obtained from the oxidation of triphenylphosphine by CP [28]. In the present study, the determination of CP was obtained from the triphenylphosphine oxide peak area. The residual peak of triphenylphosphine in the HPLC chromatograms confirmed that all CP was completely reacted. Moreover, to limit oxidative interference from other factors that might lead to an overestimation of CP, the determination of triphenylphosphine oxide in a blank sample without CP was performed, and the result is shown in Figure S1b.

### 3.1. Thermal Degradation Kinetics

**Figure 2:**
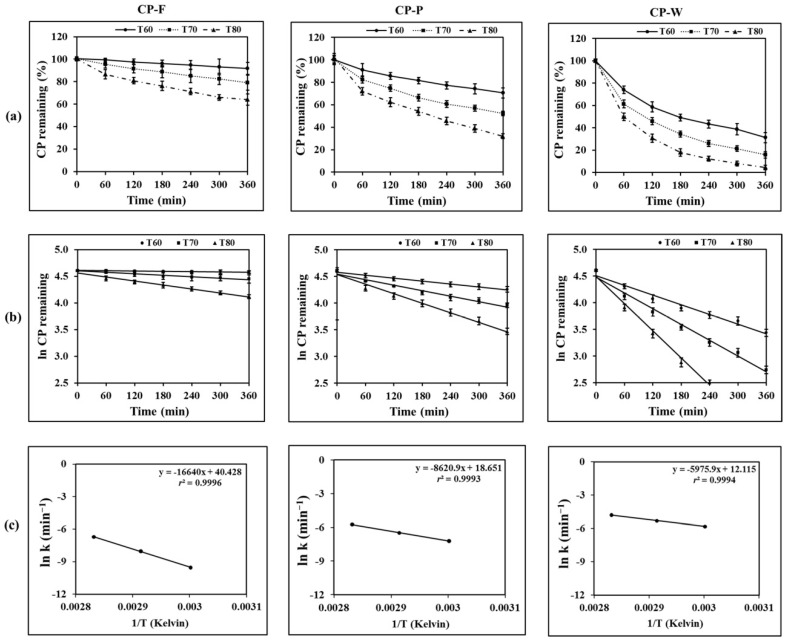
Degradation profiles of CP under heat exposure, (**a**) zero order plots, (**b**) first order plots, and (**c**) Arrhenius plots.

After thermal stress conditions, the results showed that the increase in temperature and exposure time led to an increase in CP degradation. CP content in all samples was significantly rapidly decreased from its initial value after heat exposure (*p* < 0.05). After 6 h of heating at 80 °C, the amount of CP remaining was found to be the lowest in all formulations compared to other temperatures at the same exposure time. However, the levels of CP degradation for each sample were different. CP remaining in CP-F was significantly higher (61.51 ± 0.26%) than that in CP-P (32.03 ± 2.24%) and CP-W (4.38 ± 2.16%). To evaluate the degradation kinetics of CP in the formulations, the collected experimental data were calculated based on a reaction rate expression using Equation (6):

(6) $$\frac{-d\;\left[C\right]}{\mathrm{dt}}\;=\;k{\left[C\right]}^{n},$$

where C is the concentration of CP (µg/mL), t is the incubation time (min), k is the degradation rate constant (min^−1^), and n is the order of the reaction where n = 0 is zero order and *n* = 1 is first order. The order of drug degradation was determined using graphical methods. The remaining concentration of CP and the natural logarithm remaining CP were plotted versus time for prediction of zero order and first order degradations, respectively. The results are shown in Figure 2a,b. The linear regression was added to determine the correlation coefficient (*r*^2^). As presented in Table 1, the obtained *r*^2^ values from the first order reaction plots were close to 1, suggesting that the thermal degradation of CP followed the first order kinetics. The kinetic parameters obtained from fitting the first order kinetics model are shown in Table 2. The results demonstrate that the elevated temperature could significantly cause an increase in the degradation rate of CP. The results also confirm that CP in CP-F possessed significantly higher stability than in CP-P and CP-W (*p* < 0.05).

The acceleration effect of temperature on the rate of chemical reactions is generally described by the Arrhenius equation [29], which is the relationship between the rate constant and temperature, as shown in Equation (7):

(7) $${k\;=\;\mathrm{Ae}}^{-\mathrm{Ea}/\mathrm{RT}},$$

where k is the reaction rate constant of first order kinetic (min^−1^), A is the frequency factor, Ea is the activation energy (cal mol^−1^), R is the gas constant (1.987 cal mol^−1^K^−1^), and T is the absolute temperature in degrees Kelvin. The determination of the Arrhenius parameter is based on a plot of the natural logarithm of k against the reciprocal of absolute temperature (1/T). Estimation of the appropriate rate or rate constant for CP degradation is an important step in predicting the stability of CP in each formulation. From the results, the Arrhenius plots provide a good description of CP degradation, as it is evident from the linearity (*r*^2^ = 0.99) of the plots as seen in Figure 2c for all formulations. The Ea value for CP degradation in each formulation was calculated according to the Arrhenius plots. It was found that the Ea value of CP in CP-F was higher than that in CP-P and CP-W, with the values of 33.06 ± 0.83, 17.01 ± 0.69, and 11.87 ± 0.49 kcal/mol, respectively. The results suggested that the activation energy for CP degradation in the nanofibrous film is approximately two times higher than CP in the polymer solution and three times higher than CP in the water solution. These results show the high potential of the nanofibrous film for the protection of CP from thermal degradation.

According to the Arrhenius plots, the degradation rate constant to room temperature (k_25_) of CP from each formulation can be estimated. It was found that the k_25_ of CP in CP-F was approximately 2.1 × 10^–7^ min^−1^ and that in CP-P and CP-W it was 3.5 × 10^–5^ and 36.0 × 10^–5^ min^−1^, respectively. The obtained k_25_ values were used for the calculation of the half-life and shelf-life of CP-F using Equations (8) and (9), respectively:

(8) $$t_{1/2}\;=\;\frac{0.693}{k_{25}},$$

and

(9) $$t_{90}=\;\frac{0.105}{k_{25}},$$

where (t_1/__2_) is the half-life and t_90_ is the shelf-life of CP-F. It was found that the half-life of CP-F was 6.5 ± 0.2 years, much higher than that of CP-P and CP-W, which showed the half-life values of 13.8 ± 0.8 and 1.3 ± 0.2 days, respectively. The shelf-life was calculated to ensure that at least 90% of CP remain in the formulation and the results demonstrated that the shelf-life of CP-F was 1.01 ± 0.03 years, much higher than that of CP-P and CP-W, which demonstrated shelf-life values of only 50.2 ± 2.33 (approximately 2 days) and 4.71 ± 0.63 h, respectively. From these results, the effects of nanofibrous film on retardation of CP degradation from the thermal environment was obviously seen.

### 3.2. Degradation Kinetics of CP by UV Light

**Figure 3:**
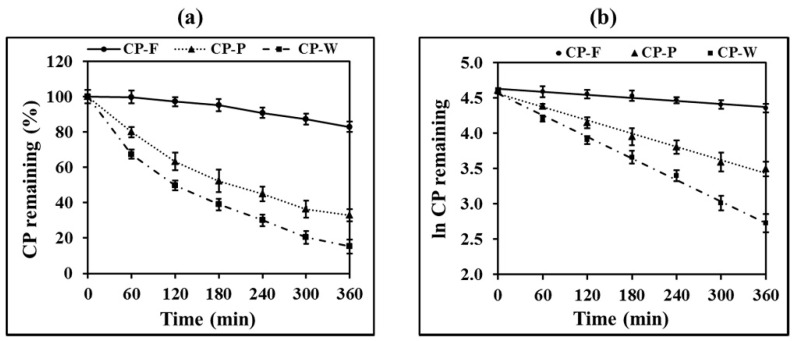
Degradation profiles of CP under UV light exposure, (**a**) zero order plots and (**b**) first order plots.

The evaluation of the photostability of the drugs and the formulations is an essential issue for formulation development. Tooth whitening agents such as hydrogen peroxide and CP are photosensitive agents [30,31]; therefore, their formulated products may degrade during manufacturing and storage. In the present study, the photostability tests of the formulations were carried out under UV light. As shown in Figure 3a, after the samples were exposed to UV light for 1 h, CP-F showed a higher proportion of CP remaining than CP-P and CP-W. The CP content of CP-P and CP-W significantly decreased when compared to the initial measurement (*p* < 0.05), whereas that of CP-F was not significantly different from the initial measurement. The CP remaining of all formulations showed a significant difference from the initial measurement (*p* < 0.05) after 4 h exposure to UV light.

The degradation profiles of CP under UV light exposure are shown in Figure 2. Plotting the data according to the first order degradation, the linear relationship was obtained as shown in Figure 3b, with the *r*^2^ close to 1, as shown in Table 3. From these results, the reaction rate constant of CP in CP-F was shown to be significantly lower than that in CP-W and CP-P. The results demonstrate that nanofibrous film prevented the degradation of CP from UV light. It is also considered that the solid dosage form has UV protection properties significantly greater than the liquid, e.g., solution, dosage form.

### 3.3. Long-Term Stability of CP-F

According to World Health Organization [32], the recommendation of the testing condition for the long-term stability of products was 25 ± 2 °C/60 ± 5% RH or 30 ± 2 °C/75 ± 5% RH for a minimum period of 12 or 6 months, respectively. In this present study, the long-term storage was conducted at an average temperature of 25 ± 2 °C for a period of 12 months. To compare the effects of temperature and humidity, a high temperature of 45 °C was used. A humidity of 75% was selected from climatic zones IV and compared with the low humidity of 30%. Thus, the storage conditions of 25 °C/30% RH, 25 °C/75% RH, and 45 °C/30% RH were used for a period of 12 months. The changes in physicochemical properties, i.e., color, morphology, internal structure, molecular interaction, mechanical properties, and mucoadhesive properties were investigated. The gradual changes in CP content in CP-F kept at the three conditions were also determined.

### 3.4. Color Changes after Long-Term Storage

The color parameters of CP-F investigated by the colorimetric measurements are shown in Table 4. Initially, CP-F was white as a high value of L* was obtained. For the degree of green−red, CP-F was achromatic as the a* value was close to 0, and for the degree of blue−yellow, CP-F was slightly blue as a negative b* value was presented. The differences between the L*, a*, and b* values of CP-F stored at 25 °C/30% RH were not significant, indicating that the color of CP-F kept at 25 °C/30% RH for 12 months was not changed. The L* value of CP-F stored at 45 °C/30% RH was the lowest compared to other conditions, indicating a significant decrease in the lightness of the samples. CP-F kept at 25 °C/75% RH and 45 °C/30% RH showed a high negative a* value and a high positive b* value, indicating the green and yellow of this sample was changed. It has been reported that the nanofibrous films containing PVA can possibly change into yellow or brown after thermal decomposition [33]. Therefore, the color change of CP-F was probably due to the decomposition of PVA which was present in the films. Among various storage conditions, no significant differences in the ΔE values were detected. As the ΔE value was lower than 3, the color change was difficult to distinguish by human eye perception [34].

### 3.5. Morphology Changes after Long-Term Storage

**Figure 4:**
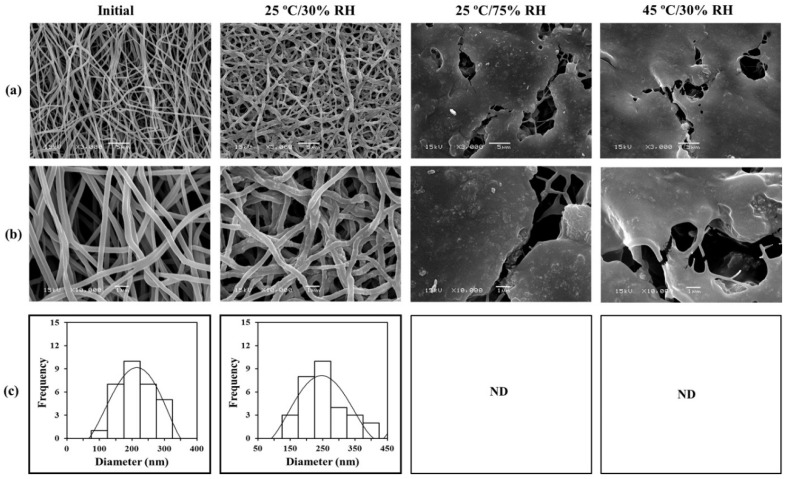
SEM images of CP-F at initial measurement and after storage at various conditions for 12 months, (**a**) at 3000× magnification, (**b**) at 10,000× magnification, and (**c**) the average diameter (ND: not detectable).

The SEM images of surface morphology and the diameter of the nanofibers in the nanofibrous films before and after keeping at the test storage conditions are presented in Figure 4. Initially, CP-F exhibited a smooth fibrous structure with a diameter range in nanosize, without any undesirable parts. After storage at 25 °C/30% RH for 12 months, CP-F showed slight defects of a straight line. However, no significant difference in average diameter was observed. The average diameters of 237 ± 57 and 267 ± 72 nm were found for CP-F at initial measurement and after storage at 25 °C/30% RH for 12 months, respectively.

After storage at 25 °C/75% RH for 12 months, CP-F exhibited different morphology from the initial measurement, and the fibrous structure was changed. Nanofibers fused together and the structure of the fibers in nanosize was almost absent. The nanofibers could not keep the original structure. These phenomena also happened with CP-F after storage at 45 °C/30% RH for 12 months. This CP-F showed the merging of the nanofibers. It was found that the remaining fibers showed discontinued and tear line structure. From these results, we considered that high temperature and high humidity are the important factors that affect the morphology of CP-F. It has been reported that high temperature can destroy the PVA-based nanofibrous film [35]. In the present study, the main composition of the nanofibers of CP-F was PVA and PVP and the results revealed that CP-F, after being subjected to high temperatures, became brittle, and the structure of the nanosized fibers was absent.

### 3.6. Internal Structure Changes after Long-Term Storage

**Figure 5:**
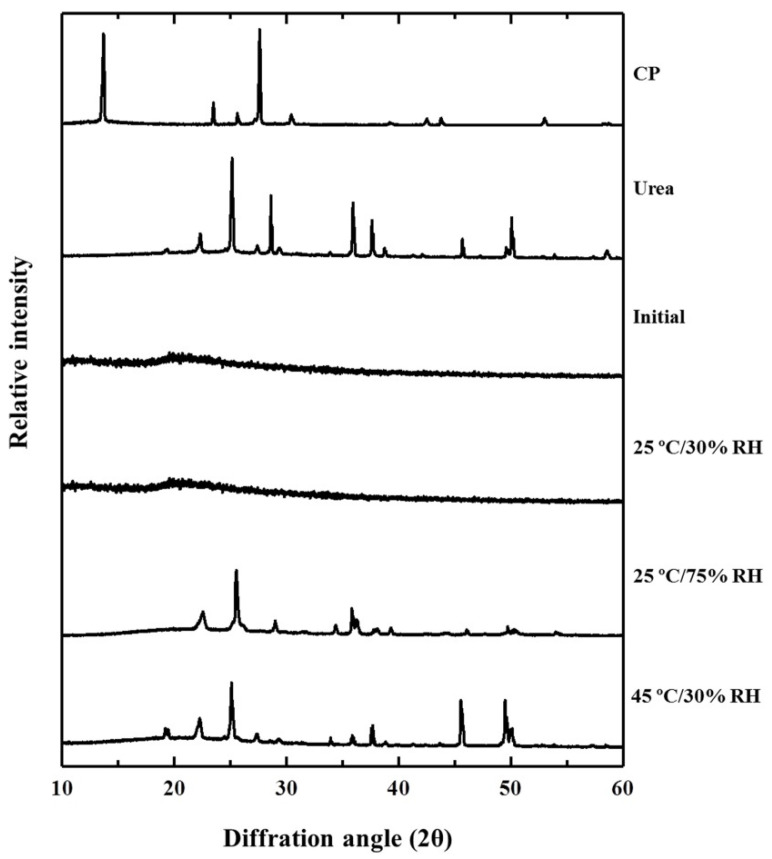
X-ray diffraction (XRD) patterns of CP-F after storage at various conditions for 12 months in comparison to the initial CP-F, CP, and urea.

The XRD patterns of intact CP and CP-F before and after storage are displayed in Figure 5. Intact CP exhibited sharp identical peaks at 14°, 23°, and 28°, indicating that the internal structure of CP was a crystalline form. The crystalline peaks of CP were absent in the XRD pattern of the freshly prepared CP-F. The disappearance of the CP crystalline peaks indicated that the drug was well incorporated in the nanofibrous film by electrospinning technique, and CP was converted from a crystalline state to an amorphous state. This halo pattern was also found in CP-F after storage at 25 °C/30% RH for 12 months, indicating that CP recrystallization did not occur during a long-term period of storage in this condition.

However, the XRD patterns of the stored CP-F under 25 °C/75% RH and 45 °C/30% RH showed high identical crystalline peaks of CP at 22° and 25°. Moreover, the XRD peaks at 46°, 49°, and 50°of CP-F after storage at 45 °C/30% RH were of higher intensity than that of CP-F storage at 25 °C/75% RH. These peaks possibly related to urea as they resemble the peak patterns of urea powder. Generally, CP dissociated into hydrogen peroxide and urea [36]. The degradation products of hydrogen peroxide are oxygen and water [37] and these products may be lost during storage. Urea was the degradation product that remained in the formulation. The amorphous urea had partially recrystallized as the crystalline form under the storage conditions; hence some crystalline peaks of urea were visible. Recrystallization of drug and polymer during storage can occur [38]. Storage conditions such as temperature and humidity could trigger molecular mobility of the drug, which might accelerate the recrystallization of the amorphous drug [39,40]. Moreover, rearrangement of the amorphous state to the crystalline phase can be accompanied by the thermo-oxidation process in the solid state [41]. The results of the present study suggest that high temperature and high humidity accelerated the degradation of CP, and increased drug recrystallization.

### 3.7. Thermal Behavior Changes after Long-Term Storage

**Figure 6:**
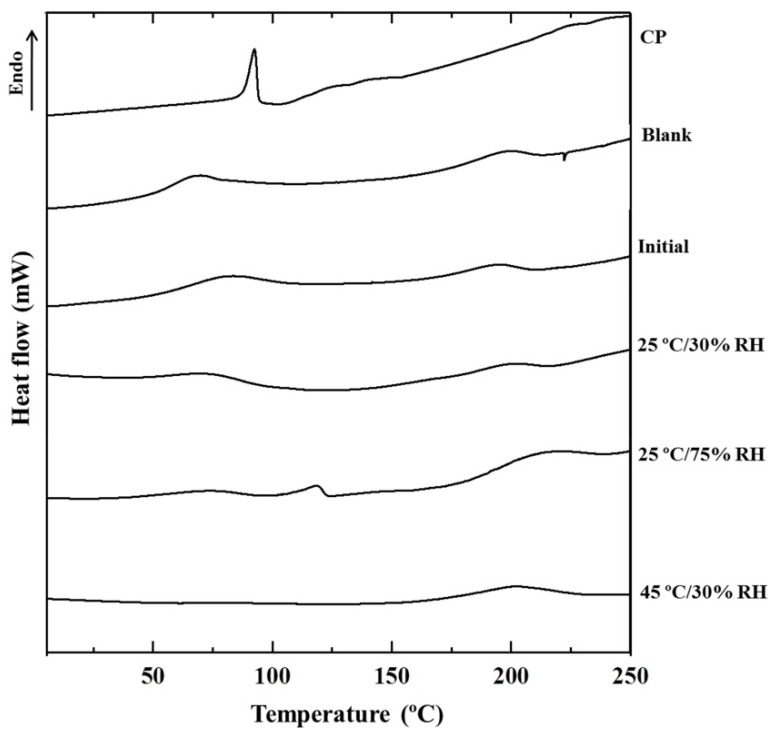
Differential scanning calorimetry (DSC) thermograms of CP-F after storage at various conditions for 12 months, in comparison to the initial CP-F, CP, and blank nanofibrous film (blank).

The thermal behavior of CP-F before and after storage in the different conditions characterized by DSC are shown in Figure 6. It was found that the DSC thermogram of intact CP displayed a sharp endothermic peak at 92 °C. Two broad endothermic curves of the blank nanofibrous film were observed at 68 and 213 °C. The DSC thermogram of CP-F showed two broad peaks similarly to the blank, however, the peaks were slightly shifted. The first endothermic broad peak of CP-F appeared at approximately 87 °C and another broad peak appeared at about 194 °C. This might be due to an interaction between CP and the excipients in the nanofibrous film. In addition, the absence of the melting CP peak in the CP-F thermogram suggested that CP was dispersed into the nanofibrous film as an amorphous form.

After long-term storage at 25 °C/30% RH, the DSC thermogram of CP-F looked similar to that of the initial state of CP-F, with no melting peaks representing the crystalline characteristics of CP or polymers observed. The results imply that the amorphous CP remained stable in the nanofibrous film. However, the endothermic peak at 118 °C was observed in the CP-F stored at 25 °C/75% RH. It has been reported that the polymer type and storage condition have a strong impact on solid-state properties [42]. PVA and PVP are hydrophilic polymers and often hygroscopic in nature: these polymers can absorb a high amount of moisture from the environment [43,44,45]. Hence, it is feasible that PVA and PVP present in CP-F would absorb water from the high humidity of 75% RH. In the meantime, degradation of CP yields hydrogen peroxide and urea, which can further break down into water and ammonia [37]. Therefore, the endothermic peak of the CP-F thermogram that appeared at 118 °C might represent the water dehydration peak of the film after sorption of the water from the degradation of CP, and the high humidity of the storage container during storage time.

### 3.8. Molecular Interaction Changes after Long-Term Storage

**Figure 7:**
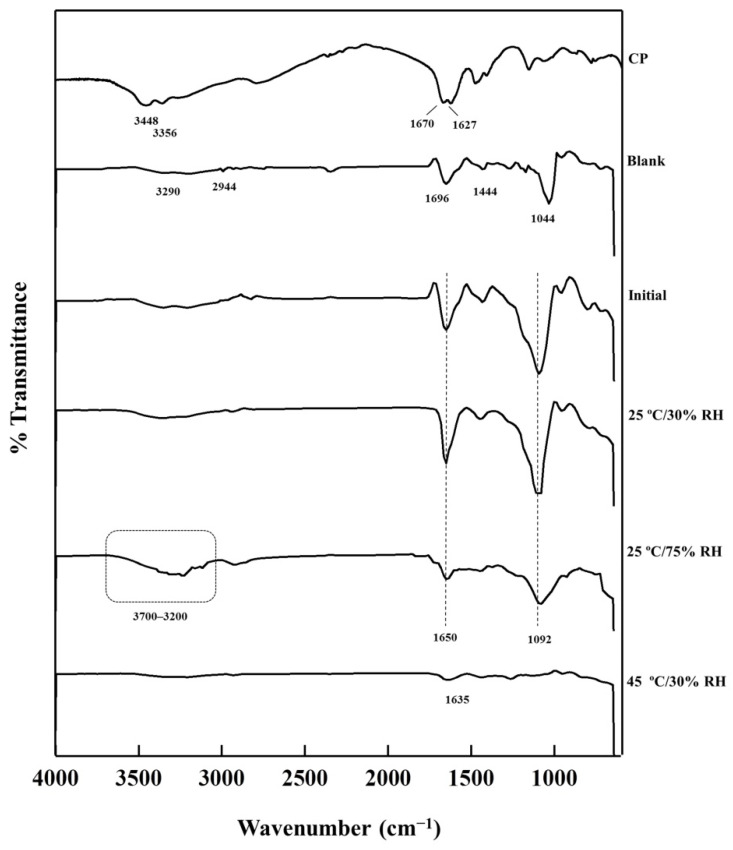
Fourier transform infrared spectroscopy (FTIR) spectrums of CP-F in the different storage conditions in comparison to the initial CP-F, CP, and blank nanofibrous film (blank).

The interaction at the molecular level between a drug and a polymer is essential to explain the stability in solid dosage forms [46]. FTIR is a useful technique for the determination of molecular interactions between drugs and polymers. Figure 7 shows the FTIR spectra of CP-F before and after storage in different conditions, obtained within the range of 4000 cm^−1^ to 600 cm^−1^. The FTIR spectrum of CP showed the band at 1670 cm^−1^ referred to C=O stretching. The bands at 1627, 3448, and 3356 cm^−1^ were corresponded to the N–H stretching of CP. The FTIR spectrum of blank nanofibrous film represented absorption peaks at 3290 cm^−1^ that referred to the O–H stretching vibration of the hydroxyl group of the base polymer. The peaks at 1444 and 2944 cm^−1^ referred to –CH_2_ bending and C–H stretching of PVA, respectively [47,48]. The absorption peaks at 1696 cm^−1^ referred to C=O from the amide group of PVP [49]. The peak around 1044 cm^−1^ was Si–O stretching [50]. The FTIR spectral pattern of CP-F was similar to that of blank nanofibrous film. The absorption peaks at around 1446–1440 cm^−1^ referred to CH_2_ bending of PVA. The weak broad band of the hydroxyl group at the 3500–3200 cm^−1^ spectral region was assigned to the O–H stretching vibration of the hydroxyl group of PVA. A low-frequency peak of the C=O stretching vibrations spectrum of PVP from 1696 to 1650 cm^−1^ was observed and a strong absorption peak at 1092 cm^−1^ was presented.

It was noted that the low-frequency of the C=O stretching vibration at 1696 cm^−1^ of PVP in the blank nanofibrous film was shifted to 1650 cm^−1^ after loading CP to the nanofibrous film. This might be due to the interaction of the peroxide and PVP [51]. In addition, the strong absorption peak at 1044 cm^−1^ was due to the siloxane bridge (Si–O–Si) of the formulations. However, after loading CP to the nanofibrous film, this peak was shifted to 1092 cm^−1^, indicating a molecular interaction with the siloxane bridge. It has been reported that hydrogen peroxide could form a strong hydrogen bond to the oxygen of the siloxane bridge [52]. The spectral shifted peak at 1092 cm^−1^ represented the interactions of hydrogen peroxide from the molecules of CP, that adsorbed on the silica surface to the siloxane bridge of the silica gel.

The FTIR spectrum of CP-F after storage at 25 °C/75% RH showed an increase in the intensity of the peak at 3700–3200 cm^−1^. As previously mentioned, the water content of CP-F could be increased due to the water sorption of CP-F during storage in high humidity, therefore the band in the region of 3700–3200 cm^−1^ was corresponded to the –OH stretching vibration of the hydrogen bonds of the water molecules [53]. However, the FTIR spectrum of CP-F after storage at 45 °C/30% RH exhibited very low intensity at the region of 3700–3200 cm^−1^, and the peak at 1092 cm^−1^ was absent. Only the stretching vibration of N–H at 1635 cm^−1^ was found. These results suggested that high temperature could lead to a decrease in the water content and hydroxyl groups [54]. Therefore, many peaks were missing due to damage from heat. Interestingly, the FTIR spectrum of CP-F after storage at 25 °C/30% RH for 12 months showed no change in the molecular interaction during the storage period. This result suggested the condition of 25 °C/30% RH was suitable for keeping CP-F.

### 3.9. Mechanical Properties Changes after Long-Term Storage

The effect of storage conditions on the mechanical properties of CP-F is of interest. The results as shown in Table 5 indicate that there was no statistically significant difference in tensile strength, elongation at break, and Young’s modulus values between initial measurements and after storage at 25 °C/30% RH. However, changes in the mechanical properties were detected in CP-F stored at 25 °C/75% RH and at 45 °C/30% RH. The higher-humidity storage led to a decrease in tensile strength and Young’s modulus value of CP-F, while the percentage of elongation at break was increased compared to the initial value. This was likely related to the water molecules in CP-F, which decrease the original interactions in the polymer matrix of the nanofibrous film [55]. Water molecules can restructure the chain networks through inter- and intramolecular hydrogen bonds [56], resulting in an increase in elongation at break and a decrease in tensile strength and Young’s modulus values. In the case of the high temperature of 45 °C/30% RH storage, the decrease in tensile strength, elongation at break, and Young’s modulus values were found. It could be noted that the higher temperature affected the strength and flexibility of the nanofibrous film, resulting in more brittle film. This result corresponds to the FTIR pattern showing the negative effect of the storage conditions on the molecular interaction of CP-F, thus, changes in mechanical properties also occurred.

### 3.10. Adhesive Property Changes after Long-Term Storage

The adhesion of the nanofibrous film is important as it affects the intended function for tooth whitening. The freshly prepared CP-F could adhere to the surface of the mucosa and the measured adhesive force was found to be 0.79 ± 0.07 N. After storage at 25 °C/30% RH for 12 months, the formulation did not show a significant difference in the adhesive properties of the film from its initial value. The adhesive force of the stored film was 0.75 ± 0.06 N. The adhesive force of CP-F after storage at 25 °C/75% RH and 45 °C/30% RH for 12 months was decreased to 0.54 ± 0.03 N and 0.31 ± 0.05 N, respectively. It was therefore suggested that the humidity and temperature influenced the adhesive properties of CP-F.

### 3.11. CP Remaining after Long-Term Storage

**Figure 8:**
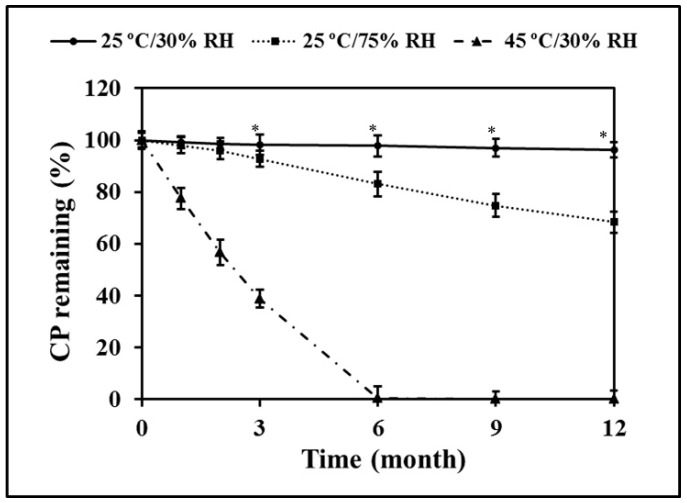
CP remaining in CP-F during long-term storage in the various conditions. * Significant difference in the amount of CP in comparison to other storage conditions (*p* < 0.05).

The stability of CP during long-term storage under different conditions is presented as degradation profiles, as shown in Figure 8. After storage for 12 months at 25 °C/75% RH and 45 °C/30% RH, CP content was significantly decreased from the initial value (*p* < 0.05). However, CP in CP-F kept at 25 °C/30% RH showed significantly higher stability than that kept at the other storage conditions. A slight reduction in CP was observed, without a significant difference in CP content between time intervals. At the end of the test period of 12 months, the remaining CP content in this condition was found to be up to 96.23 ± 3.05%, followed by that kept at 25 °C/75% RH (68.37 ± 4.17%). Stored at 45 °C/30% RH, CP could not be found after 6 months had passed, suggesting that all CP might have been completely degraded. The results also indicate that temperature had a higher effect on CP degradation than humidity.

According to the short-term stability under stress conditions of 60, 70, and 80 °C as mentioned above, the calculated shelf-life of CP in CP-F, obtained from the predicted degradation rate of Arrhenius plots at 25 °C, is approximately 1 year. This result is in agreement with the actual measured value of CP in CP-F stored at 25 °C/30% RH. However, at 25 °C/75% RH, the results show that CP degradation occurred after 3 months. This result indicates that the presence of humidity in the environment can increase the CP degradation rate.

From these results, it is suggested that the most suitable condition for keeping CP-F is that of low temperature and low humidity. In storage at high temperature, the loss of CP was increased. This can influence the efficacy of the product, leading to a lower clinical efficacy than expected. Moreover, the tooth whitening treatment would likely fail to achieve the desired outcome. The temperature and humidity, as described in the present study, played an important role in CP concentration, as well as in the performance of the nanofibrous film to deliver CP to the teeth. Therefore, the formulation should be stored in a proper condition to ensure a satisfactory clinical response. Furthermore, the formulation should be carefully packed in a humidity-impermeable container to provide a permanent barrier to protect the drug from degradation caused by humidity.

## 4. Conclusions

The degradation kinetics of CP in the prepared CP-F, CP-P, and CP-W followed the first order reaction. CP in CP-F possessed significantly higher stability than CP-P, and CP-W. The half-life of CP in CP-F was 6.5 ± 0.2 years, much higher than that of CP-P (13.8 ± 0.8 days) and CP-W (1.3 ± 0.2 days). Moreover, the nanofibrous film showed high efficiency to protect CP from light. Long-term storage of CP-F under high temperature and humidity can cause a color change, destroy the structure of nanofibers, and decrease the mechanical and adhesive properties of CP-F, as well as increase the chemical degradation of CP. High humidity enhances water absorption of CP-F leading to the degradation of CP. Among the three storage test conditions, the storage condition of 25 °C/30% RH was the most suitable for stabilizing CP-F. In conclusion, the results of the present study suggest that loading CP in a nanofibrous film and storage in the suitable conditions of low temperature and low humidity can potentially enhance CP stability.

## Figures and Tables

**Table 1:** The *r*^2^ values of a linear regression from zero and first order plots of carbamide peroxide (CP) degradation by heat exposure.

Temperature (°C)	Zero Order			First Order		
	CP-F	CP-P	CP-W	CP-F	CP-P	CP-W
60	0.95	0.95	0.90	0.99	0.99	0.98
70	0.95	0.93	0.86	0.99	0.98	0.99
80	0.94	0.91	0.78	0.98	0.98	0.99

**Table 2:** Average thermal degradation rate constant of CP in each formulation estimated by the first order kinetics.

Temperature (°C)	k (×10^−4^ min^−1^) *			
	CP-F	CP-P		CP-W
60	0.72 ± 0.13 ^c^		7.36 ± 1.21 ^b^	30.47 ± 1.58 ^a^
70	3.25 ± 0.84 ^c^		15.14 ± 1.80 ^b^	49.17 ± 1.78 ^a^
80	12.23 ± 1.49 ^c^		31.54 ± 3.49 ^b^	82.33 ± 3.14 ^a^

* Different letters within the same temperature condition are significantly different (*p* < 0.05).

**Table 3:** First order parameters of CP degradation under UV light exposure.

Formulation	Parameters	
	Rate Constant (×10^−4^ min^−1^)	*r* ^2^
CP-F	6.88 ± 0.48 ^c^	0.96
CP-P	30.36 ± 3.40 ^b^	0.99
CP-W	50.93 ± 2.95 ^a^	0.99

* Different letters are significantly different (*p* < 0.05).

**Table 4:** Color parameter of CP-F at initial measurement and after storage at various conditions for 12 months.

Conditions	Color Parameter *			
	L *	A *	B *	ΔE
Initial	55.15 ± 0.93 ^a^	−0.08 ± 0.05 ^a^	−2.17 ± 0.06 ^c^	-
25 °C/30% RH	55.69 ± 0.87 ^a^	−0.07 ± 0.04 ^a^	−2.01 ± 0.08 ^c^	1.03 ± 1.00 ^a^
25 °C/75% RH	55.92 ± 0.26 ^a^	−0.18 ± 0.03 ^b^	−1.18 ± 0.03 ^a^	1.56 ± 0.68 ^a^
45 °C/30% RH	52.61 ± 0.39 ^b^	−0.30 ± 0.06 ^c^	−1.66 ± 0.10 ^b^	2.21 ± 0.64 ^a^

* Different letters are significantly different (*p* < 0.05) for color parameter at the different conditions.

**Table 5:** Mechanical properties of CP-F at initial measurement and after storage at various conditions for 12 months.

Conditions	Tensile Strength (MPa) *	Elongation (%) *	Young’s Modulus (MPa) *
Initial	2.46 ± 0.21 ^a^	40.43 ± 3.56 ^b^	6.08 ± 0.51 ^a^
25 °C/30% RH	2.17 ± 0.18 ^a^	35.96 ± 5.37 ^b^	6.03 ± 0.51 ^a^
25 °C/75% RH	1.09 ± 0.17 ^b^	52.97 ±3.11 ^a^	2.05 ± 0.33 ^b^
45 °C/30% RH	0.28 ± 0.02 ^c^	13.01 ± 1.99 ^c^	2.15 ± 0.23 ^b^

* Different letters are significantly different (*p* < 0.05) for mechanical properties at the different conditions.

## Data Availability

Data are available upon request to the corresponding author.

## Supplementary Materials

The followings are available online at https://www.mdpi.com/article/10.3390/pharmaceutics13040449/s1, Figure S1: HPLC chromatogram of (a) triphenylphosphine oxide and residual of triphenylphosphine after oxidation by CP and (b) HPLC chromatogram of triphenylphosphine.
