# Supplementary material for: Stability and Influence of Storage Conditions on Nanofibrous Film Containing Tooth Whitening Agent
Source: Pharmaceutics. 2021 Mar 26;13(4):449. doi: 10.3390/pharmaceutics13040449 (PMC8066980; doi:10.3390/pharmaceutics13040449)
Supplement: Supplementary file 1 [file pharmaceutics-13-00449-s001.pdf]

pharmaceutics

Supplementary Materials: Stability and Influence of Storage Conditions on Nanofibrous Film Containing Tooth Whitening Agent

Siriporn Okonogi, Adchareeya Kaewpinta and Pisaisit Chaijareenont

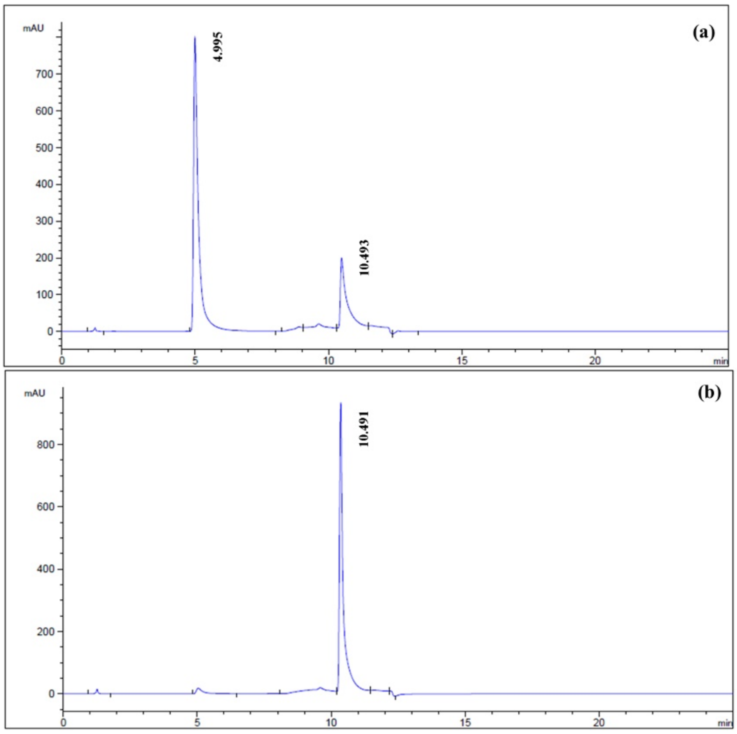

Figure S1. HPLC chromatogram of (**a**) triphenylphosphine oxide and residual of triphenylphosphine after oxidation by CP and (**b**) HPLC chromatogram of triphenylphosphine.
